# Differences of Key Proteins between Apoptosis and Necroptosis

**DOI:** 10.1155/2021/3420168

**Published:** 2021-12-12

**Authors:** Min Yeong Park, Sang Eun Ha, Preethi Vetrivel, Hun Hwan Kim, Pritam Bhangwan Bhosale, Abuyaseer Abusaliya, Gon Sup Kim

**Affiliations:** Research Institute of Life Science and College of Veterinary Medicine, Gyeongsang National University, Gazwa, Jinju 52828, Republic of Korea

## Abstract

Many different types of programmed cell death (PCD) have been identified, including apoptosis and necroptosis. Apoptosis is a type of cell death that is controlled by various genes. It is in charge of eliminating aberrant cells such as cancer cells, replenishing normal cells, and molding the body as it develops. Necroptosis is a type of programmed cell death that combines necrosis and apoptosis. In other words, it takes on a necrotic appearance, although cells die in a controlled manner. Various investigations of these two pathways have revealed that caspase-8, receptor-interacting serine/threonine-protein kinase 1 (RIPK1), and RIPK3 are crucial proteins in charge of the switching between these two pathways, resulting in the activation or inhibition of necroptosis. In this review, we have summarized the key proteins between apoptosis and necroptosis.

## 1. Introduction

Cell death has two types: unpredictable cell death and programmed cell death. Programmed cell death (PCD) refers to cell death that occurs throughout development and is not accidental [1]. PCD results in either lytic or nonlytic morphology depending on the signaling pathways involved [2].

Apoptosis, autophagy, necroptosis, pyroptosis, and other PCDs have comparable forms and kinds. Eukaryotic cells commit suicide by apoptosis, which is a highly conserved mechanism [3]. Autophagy is an intracellular evolutionarily conserved catabolic degradation process [4]. Necroptosis is a necrotic programmed cell death that has a high immunogenicity and interacts with autophagy and apoptosis [5]. Pyroptosis is an activation of inflammatory caspases that causes a lytic kind of inflammatory cell death [6].

Many studies have been conducted regarding PCD routes in recent years. The purpose of this review is to summarize the primary mechanism involved in apoptosis and necroptosis and among PCD and analyze proteins involved in each pathway. And in conclusion, another purpose is to understand the relationship between these pathways which will be helpful in the cancer disease research.

## 2. Apoptosis

Apoptosis causes a cell to stop growing and dividing. It is a process that causes cell's contents to flow into its surrounding environment [7]. Apoptosis is characterized by membrane blebbing, nuclear chromatin condensation, cell shrinkage, DNA breakdown into nucleosomal units, and the formation of apoptotic bodies [8]. Apoptosis can be triggered by either extracellular death receptors (such as tumor necrosis factor (TNF), TNF-related apoptosis-inducing ligand (TRAIL), and Fas cell surface death receptor (FAS)) or intracellular stimuli (such as nutrient deprivation, irreparable genetic damage, severe osmotic stress, and hypoxia) [1].

The extrinsic pathway (Table 1) includes connections mediated by transmembrane death receptors, TNF receptor, and extrinsic pathway and possesses genes of the superfamily [1]. Binding between death receptors and ligands will initiate this pathway, resulting in the development of a “death-included signaling complex” (DISC) consisting of TNF receptor 1 (TNFR1), Fas-Fas-L, death receptor 3 (DR3), death receptor 4 (DR4), and tumor necrosis factor superfamily 10 (known as TRAIL/Apo2L) [7].

The extrinsic route has various death receptors and ligands. We will focus on Fas-L and TNF receptors as examples. FAS will form a trimer when it is mixed with Fas-L. As a result, the FAS death effector domain, which can be coupled to the adaptor protein FAS-associated death domain (FADD), is exposed. To generate an apoptosis-inducing signal complex, procaspase-8 and procaspase-10 can attach to this domain, cleaving these procaspases to activate caspase-8 and caspase-10 which then trigger caspase-3 and caspse-7 as effector caspases, thus breaking the target protein and causing apoptosis [7].

RIPK1 and the TNF receptor- (TNFR-) associated death domain (TRADD) are also adaptor proteins linked to the cytoplasmic portion of the TNA receptor to create apoptosis complex I when the TNF trimer binds to the TNF receptor trimer [7]. Complex I was the first complex found in the respiratory system's components with a mechanistic explanation for its involvement in inducing apoptosis [9]. In the absence of proteins (such as cIAP and FLIP) that suppress a natural cell death, complexes such as RIP1, FADD, and caspase-8 will assemble in the cytoplasm, resulting in apoptosis via caspase chain reaction (apoptosis complex II) [7, 9].

The intrinsic pathway (Table 1), also known as the mitochondrial apoptosis pathway, involves various stimuli that operate on a variety of cell targets. It is caused by intrinsic lethal stimuli such as hypoxia, endoplasmic reticulum (ER) stress, metabolic stress, and DNA damage. Substances secreted by the mitochondria can trigger this type of apoptosis linked to the extrinsic pathway [7]. In extrinsic pathway, caspase-8 and caspase-10 are capable of cleaving BH3-interacting domain death agonist (BID) to truncated BID (tBID). BAX and BCL2-antagonist/killer (BAK) are activated by tBID.

Inherent fatal stimuli can stimulate BH3-only proteins which in turn activates BAX and BAK. Activated BAX and BAK can form mitochondrial permeability transition (MPT) pores on mitochondrial outer walls, allowing cytochrome c to release into the cytoplasm due to mitochondrial outer membrane permeability (MOMP) induction and act as a signaling molecule (proapoptotic proteins, including Smac/Diablo, cytochrome c, and HrtA2/Omi) in the cytoplasm to facilitate the formation of apoptosome with adapter protein apoptotic protease activating factor 1 (APAF1) in the cytoplasm. When an apoptosome is produced, caspase-9 is triggered, followed by cascades that activate caspase-3 and caspase-7. Activated caspase-3 and caspase-7 will lead to the destruction of cellular components through apoptosis [1, 7].

In addition, ripoptosome formation has been linked to the apoptotic pathway [10]. Ripoptosome is a 2 MDa protein complex that includes caspase-8, FADD, several cFLIP isoforms, and RIPK1 as key components. It can be produced by a range of cellular circumstances such as DNA damage, genotoxic stress, and IAP depletion by medicine such as teniposide/etoposide and SMAC mimetics. Teniposide is a semisynthetic phospholotoxin derivative that has similar mechanisms of action, effects, and toxicity to etoposide. Furthermore, both medicines have the property of blocking DNA synthesis in the premitotic stage of cell division by inhibiting the topoisomerase II enzyme. And they are combined with anticancer treatments. Therefore, we think that it is good to mention the substance used in combination with anticancer drugs as an example.

Flavonoids have been used in several studies to cure cancer by triggering apoptosis. Scutellarein can activate the Fas-mediated extrinsic apoptotic pathway in Hep3B cells [14]. Apigenin promotes cell death in human breast cancer MDA MB-231 and MCF-7 cells, resulting in considerable toxicity and, most importantly, apoptosis [25]. GL-V9 also induces apoptosis of human breast cancer cell lines [26]. Isoliquiritigenin, a flavonoid, can similarly stop melanoma cells from proliferating and migrating by inhibiting miR-27a expression [1, 27].

## 3. Necroptosis

Necroptosis is a type of controlled cell death that has characteristics of both apoptosis and necrosis [28]. In other terms, it is an inflammatory-mediated cell death or programmed form of cell necrosis.

As previously stated, apoptosis involves a set of mechanisms that can result in cell death. The death of cells or tissues caused by pathogenic infection, cellular injury, or noxious stimuli refers to necrosis. In another sense, necrosis is an uncontrolled and unrestricted type of cell death [8]. Necrosis occurs quickly as a result of extreme physicochemical stress such as mechanical stress, heat, osmotic shock, cell freeze-thawing, and acidity [8, 29]. Loss of plasma membrane integrity, increased cell volume, organelle swelling, lack of internucleosomal DNA breakage, and cellular collapse are all symptoms of necrosis [8]. Overexpression of certain proinflammatory proteins, such as nuclear factor kappa B, promotes cell membrane rupture and leaking of cell contents into the surrounding regions, culminating in a cascade of inflammation and tissue damage [7].

Necroptosis (Table 2) is a kind of controlled cell death characterized by morphology of necrosis. It is dependent on RIPK3 and mixed lineage kinase domain-like proteins (MLKL) [30]. It is characterized morphologically by swelling of organelles, increased cell volume, cellular collapse, permeabilization of the plasma membrane, and release of cellular contents [8].

Necroptosis induction is the activation of serine/threonine kinase RIPK1 via a cascade of signaling pathways involving the tumor necrosis factor (TNF) receptor superfamily, T cell receptors, interferon receptors, Toll-like receptors (TLRs), cellular metabolic and genotoxic stresses, or various anticancer agents that induce necroptosis [1, 12, 30, 31]. Additionally, necrostatin-1 (Nec-1), a particular necroptosis inhibitor, inhibits necroptosis as demonstrated by decreased necrotic ultrastructural changes and lower expression levels of RIPK1, RIPK3, phosphorylated MLKL, PGAM5, DRP1, and cytoplasmic HMGB1 [32, 33].

In TNFR1 signaling, TNF activates TNFR1 and causes the recruitment of RIP1 kinase, TRADD, TRAF2, and cIAP1/2, producing a transient complex known as complex I [1, 34]. TNF stimulates RIPK1 and the cellular inhibitor of apoptosis protein 1 (cIAP1), which phosphorylates the inhibitor of the kappa B kinase (IKB) complex, resulting in the activation of nuclear factor kappa B (NF-*κ*B) which leads to cell survival [35]. RIPK1 is modified by enzyme cylindromatosis (CYLD) in complex I, resulting in complex II which includes FADD, RIPK1, TRADD, and caspase-8 as key components. This step determines whether complex II leads to apoptosis or necroptosis. Inhibition of cIAP1 causes the formation of complex IIa which stimulates the caspase cascade and induces apoptosis [1]. RIPK1 and RIPK3 will form necrosome/complex IIb, a cytoplasmic necroptotic protein complex structure, if caspase-8 activity is suppressed [1, 33]. The necroptotic signal transduction pathway is triggered by complex IIb. Upon formation of complex IIb, oligomerization of mixed lineage kinase domain-like protein (MLKL) occurs, which is a downstream effector of necroptosis that initiates the necroptotic process [1, 35].

The development of numerous human diseases, including cancer, has been related to necroptosis [1]. Apigenin causes ROS-dependent necroptotic cell death through mitochondrial dysfunction due to ATP deficiency [39]. Prunetin (PRU) can induce necroptosis in a gastric cancer cell line [40]. Flavonoids can cause cancer cells that are resistant to apoptosis to undergo necroptosis [33].

## 4. Comparison of Proteins in Both Apoptosis and Necroptosis Pathways

**Figure 1:**
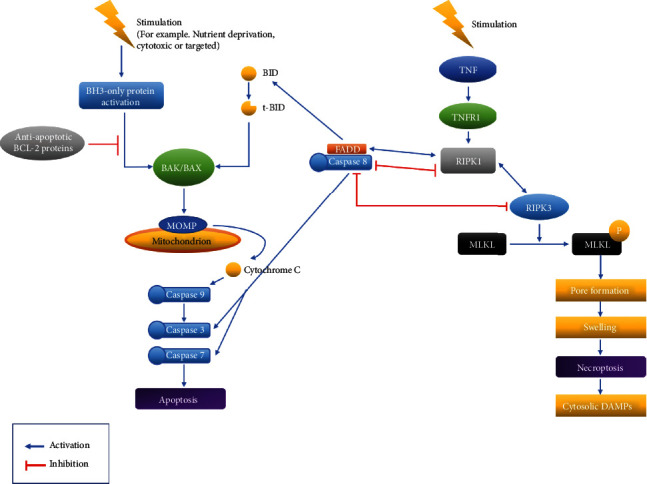
Apoptosis and necroptosis pathways. Among the pathways of apoptosis and necroptosis, activation and inhibition are explained with a focus on caspase-8.

Necroptosis is an immunologically silent inflammatory form of controlled necrotic cell death [41]. Controlled necrotic cell death has recently been discovered in several forms, all of which have morphological characteristics such as increased cellular volume, organelle swelling, and plasma membrane rupture. They have diverse triggers that involve various metabolic processes [41]. In terms of apoptosis and necroptosis, “caspase-8” is a protein that is associated with both apoptosis and necroptosis (Figure 1). Caspase-8 is an extrinsic apoptosis initiator caspase that suppresses necroptosis mediated by RIPK3 and MLKL [42]. Aside from its functions in apoptosis and necroptosis, new *in vitro* investigations have shown that caspase-8 is a scaffolding protein that can initiate cytokine synthesis independently of its enzymatic activity [42]. The scaffolding function of caspase-8 has also been implicated in activating inflammasome in macrophages triggered by double-stranded RNA (dsRNA) [42]. In addition, independent of cell death, the activity of enzyme caspase-8 is essential for NF-*κ*B activation and cytokine secretion in response to activated antigen receptors, Fc receptors, or Toll-like receptors (TLRs) [42].

When comparing the relationship of two-concerning caspase-8 between apoptosis and necroptosis, RIPK1 and RIPK3 are also involved (Figure 1). Upstream pathways activate RIPK3 by interacting with three other proteins in the mammalian genome that have conserved RIPK homotypic interaction motifs through RIPK homotypic interaction motif-dependent protein-protein interactions: ZBP1/DAI, TRIF, and RIPK1 [41]. RIPK1 is the link between RIPK3 and death receptor signaling. TLR3 and TLR4 are activated by TRIF, which then activates RIPK3. ZBP1/DAI primarily activates RIPK3 in response to viruses [41].

By functioning as a signaling hub within the apoptotic and necroptotic cell death pathways, RIPK1 controls cell survival, cell death, and inflammation. It possesses activities that are both kinase dependent and kinase independent [41]. RIPK1 can form a complex with FADD and caspase-8, in line with a previously reported signaling axis that interacts with FADD and caspase-8 to increase cell death [43, 44]. The amino acid Asp325 of RIPK1 is required for avoiding aberrant cell death in response to TNF, indicating that caspase-8 cleavage of RIPK1 is a method of disassembling death-inducing complexes [44]. Furthermore, irrespective of its kinase activity, RIPK1 functions as a scaffold to inhibit apoptosis and necroptosis in many tissues and this role is critical for avoiding inflammation and maintaining tissue homeostasis, according to several studies [41, 45, 46]. Inhibition of RIPK1 kinase activity by genetic and pharmacological means, on the other hand, has shown a critical function for RIPK1 kinase-dependent apoptosis and necroptosis in the development of inflammatory and degenerative diseases in a variety of tissues [41]. These studies have revealed that RIPK1 has opposing roles in preventing and promoting cell death, implying that its kinase-independent and kinase-dependent actions must be carefully balanced to maintain tissue homeostasis and to avoid cell death and illness [41].

Caspase-8 cleaves RIPK1 to prevent cell death and inflammation [41]. It is known that caspase-8 can inhibit necroptosis but can activate apoptosis [42]. RIPK1, RIPK3, cFLIP, and CYLD are among the proteins that caspase-8 can cleave to regulate necroptosis [41]. According to a research, Asp325 in RIPK1 is essential for avoiding abnormal cell death throughout development as well as TNF-induced cell death in a variety of cell types. Caspase-8 can cleave Asp325 of RIPK1 in cell-death signaling complexes, causing the complexes to disassemble and the death signal to be terminated [44].

**Figure 2:**
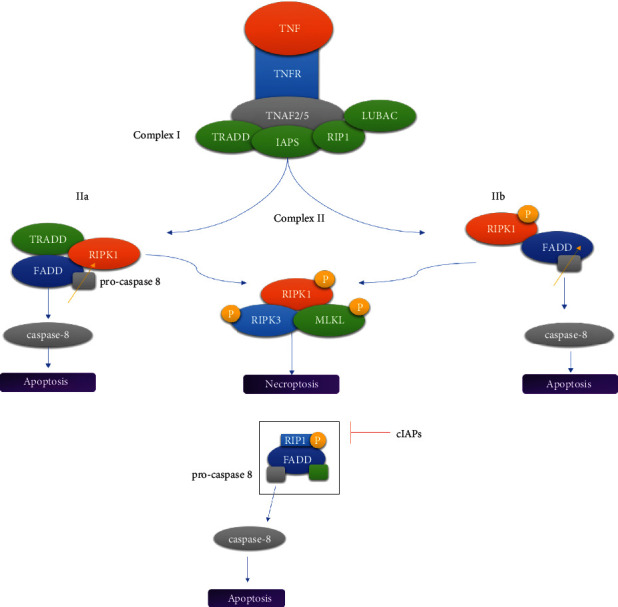
Necrosome and ripoptosome. (a) When complex I is activated by stimulation, complex II is formed and RIPK1 is dissociated. (b) When cIAPs block the ripoptosome, RIPK1 is isolated.

RIPK1 dissociates from complex I and forms either complex IIa (Ripoptosome) or complex IIb when ubiquitin carboxyl-terminal hydrolase (CYLD) (a Lys63-deubiquitylating enzyme) or cIAPs block it (necrosome) (Figure 2). When deubiquitylated RIPK1 interacts with FADD-caspase-8-FLIPL via their DDs, complex IIa is produced [46]. When RIPK1 recruits RIPK3 via their mutual RIPK homotypic interaction motifs, the riptosome is complete (RHIM). RIPK1 and RIPK3 can blanket the ripoptosome and enhance caspase-8-mediated apoptosis by having a large number of caspase-8 cleavage at sites. When caspase-8 is present as a homodimer, it is fully digested, resulting in apoptosis. When caspase-8 forms heterodimers with FLIPL, its activity toward RIPK1 is unaffected but its activity toward other apoptotic substrates including pro-caspase-3 and BID is decreased [47, 48]. As a result, despite the disassembly of the ripoptosome, cells are unable to undergo apoptosis [11, 47, 48].

Caspase-8 prevents necroptosis by cleaving the proteins RIPK1 and RIPK3. Death receptors such as DNA-dependent activator of IFN (DAI) regulatory factors can produce necroptosis as an alternate type of programmed death when caspase-8 is blocked. Necroptosis, on the other hand, produces inflammation by secreting a high number of proinflammatory cytokines and molecular patterns associated with damage (DAMPs). Caspases-8 may change the balance between apoptosis and necroptosis, making it a critical regulator of the inflammatory tumor microenvironment [47–49].

## 5. Conclusion

**Figure 3:**
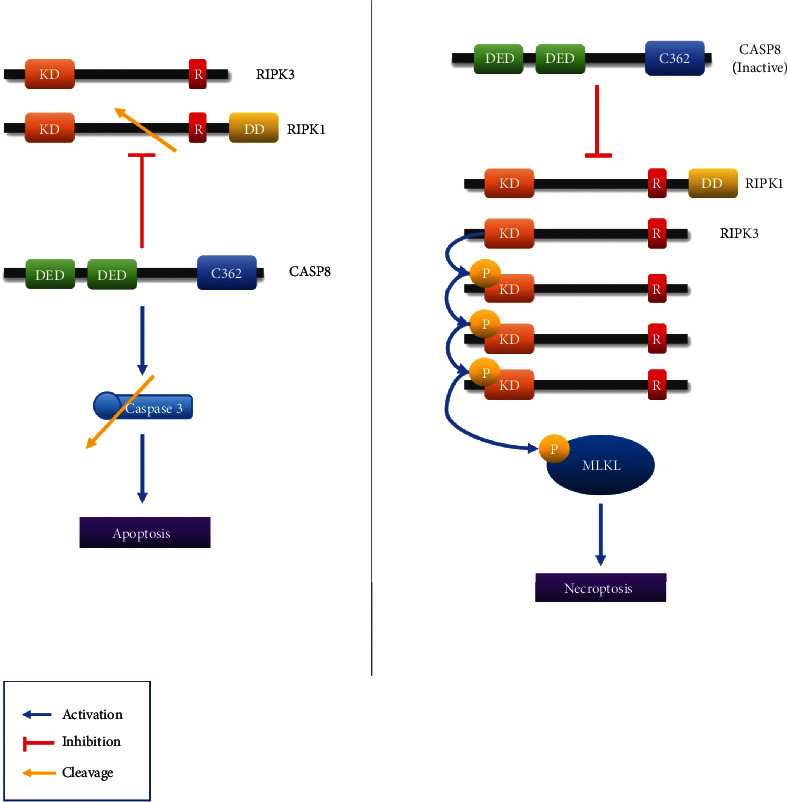
CASP8, RIPK1, and RIPK3 effect on apoptosis and necroptosis. Caspase-8 plays a role in apoptosis and necroptosis. In apoptosis, caspase-3 is cleaved to cause apoptosis, and in necroptosis, RIPK1 is inhibited to prevent necroptosis.

In this review paper, caspase-8, RIPK1, and RIPK3 are identified as key proteins involved in both apoptosis and necroptosis (Figure 3, Table 3). Apoptosis is a kind of controlled cell death marked by cell membrane blebbing, cell shrinkage, nuclear fragmentation, chromatin condensation, and chromosomal DNA breakage [12]. Necroptosis is a type of controlled cell death that is not dependent on caspases and has features that are halfway between necrosis and apoptosis [50]. We thought that it is important to examine the relationship between the two pathways. Therefore, the key proteins between these two pathways are investigated.

Caspase-8 cleaves BID to tBID during apoptosis. It has the ability to activate BAX and BAK. As a result, it activates the pathways of apoptotic mechanism. The role of caspase-8 in necroptosis is becoming more complex than its role in apoptosis (Figure 1). In necroptosis, RIPK1 and RIPK3 can inhibit caspase-8. Furthermore, RIPK1 and RIPK3 are also crucial in the transition from apoptosis to necroptosis. Caspase-8, on the other hand, is a key factor in the transition from necroptosis to apoptosis [51]. Therefore, the three key proteins involved in both apoptosis and necroptosis are intertwined to act cordially that initiates different cell death.

Overexpression of key proteins has the potential to cause illness (Table 4). These proteins are strongly linked to apoptosis and necroptosis. We think that by targeting these key proteins, therapeutic strategies for illnesses like cancer and others listed in Table 4 can be created.

The mechanisms underlying the relationship among caspase-8, RIPK1, and RIPK3 still remain to be clarified. Therefore, cell death studies targeting caspase-8, RIPK1, and RIPK3 among cell pathways have been recently conducted. We that think this review paper will provide the foundation basic knowledge for future research.

## Figures and Tables

**Table 1:** Apoptosis mechanism in cancer disease.

Protein	Up/down	Description	Article related to apoptosis	References
(1) Extrinsic pathway				
FADD	Up	The key adaptor that transmits death signals via death receptors	Induction of apoptosis in HL-60 cells by luteolin necessitates FADD-caspase-8-mediated apoptosis	[12, 13]
FasL and Fas	Up	A critical death ligand and its receptor	Treatment with SCU, on the other hand, increases expression levels of Fas and Fas ligand (FasL) known to activate cleaved caspase-3, caspase-8, and polymeric adenosine diphosphate ribose (PARP) while decreasing the expression of death receptor 4 (DR4)	[12, 14]
TRAIL	Up	TNF family death ligand	Cancer cells are destroyed whereas primary esophageal cells are protected when primary esophageal cells are cultured in a mixed population with type I cancer cells and treated with TRAIL in the presence of a caspase-9 inhibitor	[12, 15]
DR4 and DR5	Up	Death receptors for TRAIL	Casticin enhances TRAIL-induced apoptosis by downregulating cell survival proteins and inducing DR5 via ROS	[12, 16]

(2) Intrinsic pathway				
Bcl-2	Down	Regulate cell behavior through programmed cell death	The estrogenic actions of certain flavonoids may be responsible for upregulation of the Bcl2 gene in apoptotic MCF7 cells after flavonoid therapy	[17, 18]
BH3-only proteins	Up	To exert their intrinsic proapoptotic activities, all BH3-only molecules require multidomain BH3 proteins (Bax and Bak)	Phenoxodiol induces melanoma cell apoptosis by inducing p53-dependent BH3 proteins (PUMA, Noxa, and Bad) and p53-independent Bim protein, resulting in Bax activation and downstream events	[19, 20]
Bcl-**x**_**L**_	Down	Functions as apoptosis inhibitors	Fisetin, an HSF1 inhibitor, acts as a triple inhibitor, lowering expression levels of Bcl-2, Mcl-1, and Bcl-x L via downregulation of their chaperones, BAG3 and HSP70. As a result, fisetin might be beneficial in combating single agent-induced resistance	[17, 21]
BAX and BAK	Up	Results in the release of cytochrome c and activates caspases derived from mitochondria	Calycopterin treatment increases the Bax/Bcl2 ratio in HepG2 cancer cells, causing mitochondrial damage and subsequent cytochrome C release	[18, 19]
p53	Up	An important proapoptotic factor and tumor inhibitor	N101-2 treatment decreases expression levels of cyclin A and p-pRb while increasing expression levels of p53, p21, and p27	[19, 22]

(3) Caspase and caspase inhibitors				
Caspase-8	Up	Initiator caspase that promotes the activation of caspase-3	The ligand binding to the transmembrane death receptor initiates the extrinsic apoptotic pathway, which leads in caspase-8 activation	[18]
Caspase-10	Up	Activation of signal transduction cascade is initiated by a caspase initiator	Caspase-10 is cleaved in response to flavone treatment	[17, 23]
Caspase-3	Up	Caspase effector	Fisetin activates caspase-3 and caspase-7 in a dose-dependent way. Such caspase activation coincides with PARP cleavage	[21]
IAPs (XIAP, cIAP1/2)	Down	Inhibitors of apoptosis proteins	Survivin, an inhibitor of apoptosis (IAP) family member, showed a reduction in expression following DHM therapy, perhaps due to p53 activation	[24]

**Table 2:** Necroptosis mechanism in cancer disease.

Protein	Up/down	Description	Article related to necroptosis	References
TNF	Up	Tumor necrosis factor	Fisetin significantly increases TNF and IK expression while decreasing pNF-, and pIK expression	[35]
RIPK1 and RIPK3	Up	Receptor-interacting protein kinase-1 and 3	In the presence of ZVAD, MCF-7 cells express substantially more RIPK1 and RIPK3 in response to Que than in the absence of ZVAD	[33]

TNFR1	Up	TNF's receptor following the formation of two TNFR complexes	Cell survival, apoptosis, or necroptosis can result from TNFR1 stimulation caused by damage, cellular stress, or infection	[8, 36]
			TNF-*α*, TNFR1, and necroptosis protein expression are all inhibited by PPO, indicating that PPO can protect neurons by preventing TNF-*α*-induced necroptosis	[37]

FADD	Up	Fas-associated protein with death domain (FADD). Activation of MLKL	Necroptosis is caused by caspase-8, FADD, and RIPK3 (complex IIa/b)	[36]
			FADD expression in HepG2 is reduced by fisetin treatment. FADD protects intestinal epithelial cells from RIP3-induced cell necrosis	[35]

MLKL	Up	MLKL (mixed lineage kinase like) after rapid plasma membrane rupture and inflammatory response via DAMP and cytokine release	M1 CM significantly increases expression levels of MLKL, RIPK3, and p-MLKL after quercetin treatment	[36, 38]

**Table 3:** Key proteins involved in both apoptosis and necroptosis.

Protein	Relation of apoptosis and necroptosis	References
RIPK1, RIPK3	In response to TNF, RIPK1 Asp325 is needed for reducing aberrant cell death	[44]
	Inhibiting caspase-8-mediated apoptosis as well as RIPK3-MLKL-dependent necroptosis fully prevents RIPK1-mediated embryonic mortality	[41]

Caspase-8	Caspase-8 is a molecular switch that controls apoptosis and necroptosis, as well as protecting tissues from injury	[42]
	Caspase-8 cleaves itself, other proteins, or both in order to prevent necroptosis	[44]

**Table 4:** The relationship between disease and key proteins.

Key proteins	Effect in disease	Related diseases	FDA-approved inhibitor (company)	References
Caspase-8	Mediator	Inflammation and disease in rodent malaria	None	[52]
RIPK1	Activation of RIPK1	Skin diseases, including melanoma, psoriasis, and systemic lupus erythematosus	Sunitinib (Pfizer), Pazopanib (GSK, Novartis)	[53, 54]
RIPK3	Mediator	Sepsis-associated organ injury and chronic lung diseases	Dabrafenib (GSK, Novartis)	[54, 55]

## Abbreviations

PCD: Programmed cell death
RIPK1: Receptor-interacting serine/threonine protein kinase 1
TNF: Tumor necrosis factor
TRAIL: TNF-related apoptosis-inducing ligand
FAS: Fas cell surface death receptor
DISC: Death-included signaling complex
TNFR1: TNF receptor 1
DR3: Death receptor 3
FADD: FAS-associated death domain
TRADD: TNFR-associated death domain
ER: Endoplasmic reticulum
BID: BH3-interacting domain death agonist
tBID: Truncated BID
BAK: BCL-2-antagonist/killer
MOMP: Mitochondrial outer membrane permeability
APAF1: Apoptotic protease activating factor 1
FasL: Fas ligand
PARP: Polymeric adenosine diphosphate ribose
MLKL: Mixed lineage kinase domain-like proteins
TLRs: Toll-like receptors
Nec-1: Necrostatin-1
cIAP 1: Cellular inhibitor of apoptosis protein 1
IkB: Inhibitor of kappa B kinase
NF-*κ*B: Nuclear factor kappa B
DAI: DNA-dependent activator of IFN
DAMPs: Damage-associated molecular pattern.

## References

[1] Mishra A. P., Salehi B., Sharifi-Rad M. (2018). Programmed cell death, from a cancer perspective: an overview. *Molecular Diagnosis & Therapy*.

[2] Jorgensen I., Rayamajhi M., Miao E. A. (2017). Programmed cell death as a defence against infection. *Nature Reviews. Immunology*.

[3] Vermeulen K., Berneman Z. N., Van Bockstaele D. R. (2003). Cell cycle and apoptosis. *Cell Proliferation*.

[4] Li X., He S., Ma B. (2020). Autophagy and autophagy-related proteins in cancer. *Molecular Cancer*.

[5] Gong Y., Fan Z., Luo G. (2019). The role of necroptosis in cancer biology and therapy. *Molecular Cancer*.

[6] Kesavardhana S., Malireddi R. K. S., Kanneganti T. D. (2020). Caspases in cell death, inflammation, and pyroptosis. *Annual Review of Immunology*.

[7] D'Arcy M. S. (2019). Cell death: a review of the major forms of apoptosis, necrosis and autophagy. *Cell Biology International*.

[8] Belizario J., Vieira-Cordeiro L., Enns S. (2015). Necroptotic cell death signaling and execution pathway: lessons from knockout mice. *Mediators of Inflammation*.

[9] Lemarie A., Grimm S. (2011). Mitochondrial respiratory chain complexes: apoptosis sensors mutated in cancer?. *Oncogene*.

[10] Schwabe R. F., Luedde T. (2018). Apoptosis and necroptosis in the liver: a matter of life and death. *Nature Reviews. Gastroenterology & Hepatology*.

[11] Schilling R., Geserick P., Leverkus M. (2014). Characterization of the Ripoptosome and Its Components: Implications for Anti- inflammatory and Cancer Therapy. *Methods in Enzymology*.

[12] Su Z., Yang Z., Xu Y., Chen Y., Yu Q. (2015). Apoptosis, autophagy, necroptosis, and cancer metastasis. *Molecular Cancer*.

[13] Wang S. W., Chen Y. R., Chow J. M. (2018). Stimulation of Fas/FasL-mediated apoptosis by luteolin through enhancement of histone H3 acetylation and c-Jun activation in HL-60 leukemia cells. *Molecular Carcinogenesis*.

[14] Sang Eun H., Seong Min K., Ho Jeong L. (2019). Scutellarein induces Fas-mediated extrinsic apoptosis and G2/M cell cycle arrest in Hep3B hepatocellular carcinoma cells. *Nutrients*.

[15] Ozoren N., El-Deiry W. S. (2002). Defining characteristics of types I and II apoptotic cells in response to TRAIL. *Neoplasia*.

[16] Tang S. Y., Zhong M. Z., Yuan G. J. (2013). Casticin, a flavonoid, potentiates TRAIL-induced apoptosis through modulation of anti-apoptotic proteins and death receptor 5 in colon cancer cells. *Oncology Reports*.

[17] Abotaleb M., Samuel S. M., Varghese E. (2019). Flavonoids in cancer and apoptosis. *Cancers*.

[18] Moradi M., Gholipour H., Sepehri H. (2020). Flavonoid calycopterin triggers apoptosis in triple-negative and ER-positive human breast cancer cells through activating different patterns of gene expression. *Naunyn-Schmiedeberg’s Archives of Pharmacology*.

[19] Ouyang L., Shi Z., Zhao S. (2012). Programmed cell death pathways in cancer: a review of apoptosis, autophagy and programmed necrosis. *Cell Proliferation*.

[20] Yu F., Watts R. N., Zhang X. D., Borrow J. M., Hersey P. (2006). Involvement of BH3-only proapoptotic proteins in mitochondrial-dependent Phenoxodiol-induced apoptosis of human melanoma cells. *Anti-Cancer Drugs*.

[21] Kim J. A., Lee S., Kim D. E., Kim M., Kwon B. M., Han D. C. (2015). Fisetin, a dietary flavonoid, induces apoptosis of cancer cells by inhibiting HSF1 activity through blocking its binding to the hsp70 promoter. *Carcinogenesis*.

[22] Kim J. H., Kang J. W., Kim M. S. (2012). The apoptotic effects of the flavonoid N101-2 in human cervical cancer cells. *Toxicology In Vitro*.

[23] Erhart L. M., Lankat-Buttgereit B., Schmidt H., Wenzel U., Daniel H., Goke R. (2005). Flavone initiates a hierarchical activation of the caspase-cascade in colon cancer cells. *Apoptosis*.

[24] Xu Y., Wang S., Chan H. F. (2017). Dihydromyricetin induces apoptosis and reverses drug resistance in ovarian cancer cells by p53-mediated downregulation of survivin. *Scientific Reports*.

[25] Vrhovac Madunic I., Madunic J., Antunovic M. (2018). Apigenin, a dietary flavonoid, induces apoptosis, DNA damage, and oxidative stress in human breast cancer MCF-7 and MDA MB-231 cells. *Naunyn-Schmiedeberg’s Archives of Pharmacology*.

[26] Guo Y., Wei L., Zhou Y. (2020). Flavonoid GL-V9 induces apoptosis and inhibits glycolysis of breast cancer via disrupting GSK-3*β*-modulated mitochondrial binding of HKII. *Free Radical Biology & Medicine*.

[27] Xiang S., Zeng H., Xia F. (2021). The dietary flavonoid isoliquiritigenin induced apoptosis and suppressed metastasis in melanoma cells: An _in vitro_ and _in vivo_ study. *Life Sciences*.

[28] Dhuriya Y. K., Sharma D. (2018). Necroptosis: a regulated inflammatory mode of cell death. *Journal of Neuroinflammation*.

[29] Orrenius S., Nicotera P., Zhivotovsky B. (2011). Cell death mechanisms and their implications in toxicology. *Toxicological Sciences*.

[30] Galluzzi L., Kepp O., Chan F. K., Kroemer G. (2017). Necroptosis: mechanisms and relevance to disease. *Annual Review of Pathology*.

[31] Vanden Berghe T., Linkermann A., Jouan-Lanhouet S., Walczak H., Vandenabeele P. (2014). Regulated necrosis: the expanding network of non-apoptotic cell death pathways. *Nature Reviews. Molecular Cell Biology*.

[32] Liu Y., Xu Q., Wang Y. (2021). Necroptosis is active and contributes to intestinal injury in a piglet model with lipopolysaccharide challenge. *Cell Death & Disease*.

[33] Khorsandi L., Orazizadeh M., Niazvand F., Abbaspour M. R., Mansouri E., Khodadadi A. (2017). Quercetin induces apoptosis and necroptosis in MCF-7 breast cancer cells. *Bratislavské Lekárske Listy*.

[34] Vandenabeele P., Declercq W., Van Herreweghe F., Vanden Berghe T. (2010). The role of the kinases RIP1 and RIP3 in TNF-induced necrosis. *Science Signaling*.

[35] Sundarraj K., Raghunath A., Panneerselvam L., Perumal E. (2020). Fisetin, a phytopolyphenol, targets apoptotic and necroptotic cell death in HepG2 cells. *BioFactors*.

[36] Seifert L., Miller G. (2017). Molecular pathways: the necrosome-a target for cancer therapy. *Clinical Cancer Research*.

[37] Wang W., Xie L., Zou X. (2021). Pomelo peel oil suppresses TNF-*α*-induced necroptosis and cerebral ischaemia-reperfusion injury in a rat model of cardiac arrest. *Pharmaceutical Biology*.

[38] Fan H., Tang H. B., Shan L. Q. (2019). Quercetin prevents necroptosis of oligodendrocytes by inhibiting macrophages/microglia polarization to M1 phenotype after spinal cord injury in rats. *Journal of Neuroinflammation*.

[39] Lee Y. J., Park K. S., Nam H. S., Cho M. K., Lee S. H. (2020). Apigenin causes necroptosis by inducing ROS accumulation, mitochondrial dysfunction, and ATP depletion in malignant mesothelioma cells. *The Korean Journal of Physiology & Pharmacology*.

[40] Vetrivel P., Kim S. M., Ha S. E. (2020). Compound prunetin induces cell Death in gastric cancer cell with potent anti-proliferative properties: in vitro assay, molecular docking, dynamics, and ADMET studies. *Biomolecules*.

[41] Schwarzer R., Laurien L., Pasparakis M. (2020). New insights into the regulation of apoptosis, necroptosis, and pyroptosis by receptor interacting protein kinase 1 and caspase-8. *Current Opinion in Cell Biology*.

[42] Fritsch M., Günther S. D., Schwarzer R. (2019). Caspase-8 is the molecular switch for apoptosis, necroptosis and pyroptosis. *Nature*.

[43] Zhou H., Zhou M., Hu Y. (2021). TNF-*α* triggers RIP1/FADD/caspase-8-mediated apoptosis of astrocytes and RIP3/MLKL-mediated necroptosis of neurons induced by Angiostrongylus cantonensis infection. *Cellular and Molecular Neurobiology*.

[44] Newton K., Wickliffe K. E., Dugger D. L. (2019). Cleavage of RIPK1 by caspase-8 is crucial for limiting apoptosis and necroptosis. *Nature*.

[45] O’Donnell J. A., Lehman J., Roderick J. E. (2018). Dendritic cell RIPK1 maintains immune homeostasis by preventing inflammation and autoimmunity. *Journal of Immunology*.

[46] Dillon C. P., Weinlich R., Rodriguez D. A. (2014). RIPK1 blocks early postnatal lethality mediated by caspase-8 and RIPK3. *Cell*.

[47] Mandal R., Barron J. C., Kostova I., Becker S., Strebhardt K. (2020). Caspase-8: the double-edged sword. *Biochimica Et Biophysica Acta. Reviews on Cancer*.

[48] Tummers B., Green D. R. (2017). Caspase-8: regulating life and death. *Immunological Reviews*.

[49] Zhu F., Zhang W., Yang T., He S. D. (2019). Complex roles of necroptosis in cancer. *Journal of Zhejiang University. Science. B*.

[50] Negroni A., Colantoni E., Cucchiara S., Stronati L. (2020). Necroptosis in intestinal inflammation and cancer: new concepts and therapeutic perspectives. *Biomolecules*.

[51] Vanden Berghe T., Kaiser W. J., Bertrand M. J., Vandenabeele P. (2015). Molecular crosstalk between apoptosis, necroptosis, and survival signaling. *Molecular & Cellular Oncology*.

[52] Pereira L. M. N., Assis P. A., de Araújo N. M. (2020). Caspase-8 mediates inflammation and disease in rodent malaria. *Nature Communications*.

[53] Jin L., Liu P., Yin M., Zhang M., Kuang Y., Zhu W. (2020). RIPK1: a rising star in inflammatory and neoplastic skin diseases. *Journal of Dermatological Science*.

[54] Martens S., Hofmans S., Declercq W., Augustyns K., Vandenabeele P. (2020). Inhibitors targeting RIPK1/RIPK3: old and new drugs. *Trends in Pharmacological Sciences*.

[55] Choi M. E., Price D. R., Ryter S. W., Choi A. M. K. (2019). Necroptosis: a crucial pathogenic mediator of human disease. *Insight*.

